# Demographic and Social Patterns of the Mean Values of Inflammatory Markers in U.S. Adults: A 2009–2016 NHANES Analysis

**DOI:** 10.3390/diseases11010014

**Published:** 2023-01-20

**Authors:** Rose Calixte, Zachary Ye, Raisa Haq, Salwa Aladhamy, Marlene Camacho-Rivera

**Affiliations:** 1 School of Public Health, SUNY Downstate Health Sciences University, Brooklyn, NY 11203, USA; 2 College of Medicine, SUNY Downstate Health Sciences University, Brooklyn, NY 11203, USA; 3 School of Medicine, City University of New York, New York, NY 10031, USA; 4 College of Optometry, Pennsylvania State University, State College, PA 16802, USA

**Keywords:** inflammation, cancer, biomarkers, neutrophil, platelet, lymphocyte, smoking

## Abstract

Several studies have reported on the negative implications of elevated neutrophil-to-lymphocyte ratio (NLR) and elevated platelet-to-lymphocyte ratio (PLR) levels associated with outcomes in many surgical and medical conditions, including cancer. In order to use the inflammatory markers NLR and PLR as prognostic factors in disease, a normal value in disease-free individuals must be identified first. This study aims (1) to establish mean values of various inflammatory markers using a healthy and nationally representative U.S. adult population and (2) to explore heterogeneity in the mean values by sociodemographic and behavioral risk factors to better specify cutoff points accordingly. The National Health and Nutrition Examination Survey (NHANES) of aggregated cross-sectional data collected from 2009 to 2016 was analyzed; data extracted included markers of systemic inflammation and demographic variables. We excluded participants who were under 20 years old or had a history of an inflammatory disease such as arthritis or gout. Adjusted linear regression models were used to examine the associations between demographic/behavioral characteristics and neutrophil counts, platelet counts, lymphocyte counts, as well as NLR and PLR values. The national weighted average NLR value is 2.16 and the national weighted average PLR value is 121.31. The national weighted average PLR value for non-Hispanic Whites is 123.12 (121.13–125.11), for non-Hispanic Blacks it is 119.77 (117.49–122.06), for Hispanic people it is 116.33 (114.69–117.97), and for participants of other races it is 119.84 (116.88–122.81). Non-Hispanic Blacks and Blacks have significantly lower mean NLR values (1.78, 95% CI 1.74–1.83 and 2.10, 95% CI 2.04–2.16, respectively) as compared with that of non-Hispanic Whites (2.27, 95% CI 2.22–2.30, *p* < 0.0001). Subjects who reported a non-smoking history had significantly lower NLR values than subjects who reported any smoking history and higher PLR values than current smokers. This study provides preliminary data for demographic and behavioral effects on markers of inflammation, i.e., NLR and PLR, that have been associated with several chronic disease outcomes, suggesting that different cutoff points should be set according to social factors.

## 1. Introduction

Inflammatory cells have been found to play roles in a variety of chronic conditions, such as cardiovascular disease [1], chronic kidney disease [2], and cancer [3,4,5,6,7]. Hematological components of the systemic inflammatory response (SIR), also known as SIR biomarkers, are increasingly becoming potential prognostic factors of various diseases [8]. Two such inflammatory response markers that have been widely used are the neutrophil-to-lymphocyte ratio (NLR) and the platelet-to-lymphocyte ratio (PLR).

NLR is the ratio of circulating neutrophils to lymphocytes and can be calculated from a complete blood count [9]. An elevated NLR value has been associated with shorter survival in lung [3], pancreatic [4], and colorectal [5] cancers and serves as a marker of infectious pathologies and post-operative complications [6,10]. However, there is no current standard value that is considered to be a normal vs. abnormal NLR value. Current studies have defined NLR cutoff points contingent to their respective methodologies and populations. Some studies have reported NLR values organized into intervals [11,12], while other studies have chosen to define a cutoff point based on the median value calculated from the sample (NLR > 3.5) [2], and other studies have defined an elevated NLR based on poor survival in their sample (NLR > 5) [5].

Another inflammatory marker that has potential prognostic value for disease is the PLR, which is the ratio of circulating platelets to lymphocytes. An elevated PLR has been shown to be an independent prognostic factor for cardiovascular diseases, especially heart failure. It has been found that heart failure patients have both higher PLR values and higher NLR values [13]. PLR has also been associated with a worse prognosis or poorer oncological outcomes such as poorer overall survival and more advanced staging in a variety of malignancies, including gastric [14], colorectal [15], and pancreatic [16] cancers. The standard reference range for PLR is also uncertain, as this value appears to vary depending on a variety of factors [17].

It has been established that elevated NLR and PLR values are generally prognostic factors of mortality and morbidity in the diseases they are associated with. While current studies have looked at the significance of NLR and PLR in diseased populations, not much is known about the NLR and PLR values in normal, healthy populations. Having universal reference values based on a large and healthy population will allow for better use of these markers, which can lead to potential clinical significance in determining if a patient is in good health. One study tried to determine the limits of the values of NLR that were observable in an adult, non-geriatric population and identified them as 0.78–3.53 [18]. However, NLR and PLR values have been shown to differ based on demographic factors [17,19]. There has also been an association found between smoking status and NLR and PLR levels. Smoking appears to increase NLR [20] and decrease PLR [20,21]. As such, this study aims (1) to establish mean values of various inflammatory markers using a healthy and nationally representative U.S. adult population and (2) to explore heterogeneity in the mean values by sociodemographic and behavioral risk factors to better specify cutoff points accordingly.

## 2. Materials and Methods

The National Health and Nutrition Examination Survey (NHANES), a population-based survey that is designed to assess the health and nutritional status of non-institutionalized adults and children in the United States, was used for analysis. The NHANES uses a complex, multistage, probability sampling design to produce a nationally representative sample. In this study, we aggregated cross-sectional data collected from 2009 to 2016 and extracted various, validated measures of general inflammation (lymphocyte, monocyte, segmented neutrophil, eosinophil, basophil, platelet count, NLR, and PLR), demographic characteristics (age, sex, race, and body mass index), and social factors (education level, nativity to USA, smoking status, and alcohol usage).

Consenting participants complete a detailed in-person interview that is conducted by a trained professional on topics encompassing their demographic, socioeconomic, dietary, and health-related information. Measures of age, sex, race, education, nativity to USA, smoking status, and alcohol usage are obtained at that time. After an in-home interview, participants are scheduled an appointment at a Mobile Examination Center (MEC) where medical, physiologic, and laboratory tests are administered by trained medical staff [22]. At this time, body mass index is measured using bioelectrical impedance. Hematology testing is performed on blood specimens collected from participants and evaluated for neutrophil (1000 cells/µL), monocyte (1000 cells/µL), segmented neutrophil (1000 cells/µL), eosinophil (1000 cells/µL), basophil (1000 cells/µL), and platelet (1000 cells/µL) counts. Cell counts are determined using the Coulter MAXMs method (Beckman Coulter, Miami, FL, USA).

We excluded participants who were under 20 years old or had a history of inflammatory disease such as arthritis or gout. The final sample consisted of 16,849 subjects across all of the survey waves. We categorized age into four categories (20–29, 30–59, 60–79, and ≥80) to balance across sample sizes and because the NHANES has stopped reporting the actual age of anyone over 80 years old and uses 80 as a ceiling for age. We categorized BMI into four clinically important categories (underweight, normal weight, overweight, and obese).

We summarized all variables of interest using appropriate descriptive statistics such as unweighted mean (95% confidence interval) or unweighted frequency (percent) and weighted mean (95% CI) or weighted proportion (95% CI). We tested for bivariate association between demographic and behavioral characteristics on general inflammatory markers using linear regression models for each outcome. We tested for independent predictors of inflammatory markers using multiple linear regressions. We preprocessed the data using the SAS 9.4^®^ software and analyzed the data using the Stata/SE version 16 software, with appropriate complex survey design methodology to provide nationally representative estimates. Results with *p*-value ≤ 0.05 are significant.

## 3. Results

### 3.1. Mean Inflammatory Marker Values in U.S. Adults

We present summary statistics of the study population with 95% confidence intervals in Table 1. The weighted study population is 62.5% non-White Hispanics, 11.6% non-Hispanic Black, and 16.7 % Hispanics. The proportions of males and females in the population are equally distributed. Over 82% of the study population are between 20 years old and 59 years old. Over 66% of the included participants are either overweight or obese. About 32% have a college degree or more. Moreover, close to 79% of the population are U.S. born citizens. About 60% of the included participants are non-smokers and just under 12% of the participants are non-drinkers. In the weighted population, the national mean for lymphocytes (1000 cells/µL) is 2.14 (95% CI = 2.11–2.16), the national mean for monocytes (1000 cells/µL) is 0.56 (95% CI = 0.55–0.56), the national mean for neutrophils (1000 cells/µL) is 4.28 (95% CI = 4.23–4.34), the national mean for eosinophils (1000 cells/µL) is 0.20 (95% CI = 0.19–0.20), the national mean for basophils (1000 cells/µL) is 0.05 (95% CI = 0.04–0.05), the national mean for platelet count (1000 cells/µL) is 238.89 (95% CI = 237.26–240.53), the national mean NLR value is 2.16 (95% CI = 2.13–2.19), And the national mean PLR value is 121.31 (95% CI = 102.01–122.61). The national weighted average (95% CI) numbers for the inflammatory markers are within the normal range for healthy people.

### 3.2. Univariate Associations of Demographic Factors and Inflammatory Markers

We present the results of the univariate regression model for inflammatory markers in Table 2. On average, non-Hispanic Black adults have higher lymphocyte counts, higher platelet counts, lower monocyte counts, lower neutrophil counts, lower eosinophil counts, lower basophil counts, and lower NLR values than non-Hispanic Whites. However, the mean inflammatory marker counts for within racial categories are within a normal range. A gender difference was also observed within the inflammatory markers, with adult males having lower mean lymphocyte counts, lower mean neutrophil counts, lower mean platelet counts, and lower mean PLR values as compared with those of female adults. However, on average, adult males had higher NLR values, higher eosinophil counts, and higher monocyte counts than adult females in the U.S. population. The mean values for inflammatory markers were different for U.S.-born participants vs. non-U.S.-born participants. As compared with non-U.S.-born adults, U.S.-born adults had lower mean lymphocyte counts, higher mean monocyte counts, higher mean neutrophil counts, higher mean platelet counts, and higher mean NLR values. However, foreign born adults had higher PLR values as compared with U.S.-born adults. Participants with normal BMI values had the lowest mean values for lymphocyte counts and neutrophil counts, but the highest mean value for PLR. Comparing across demographic and clinical categories, the mean values for all inflammatory markers are within the normal range.

### 3.3. Multivariable Regression Results for Inflammatory Markers

We present the results of the multivariable regression model for inflammatory markers in Table 3. The results of the multivariable model reveal that, on average, lymphocyte count data are significantly higher in non-Hispanic Blacks and Hispanics as compared with non-Hispanic Whites. On average, lymphocyte counts are significantly higher for females as compared with males. Participants who were 30–79 years old had significantly lower mean lymphocyte counts vs. participants who were between 20 and 29 years old. Non-U.S.-born participants in the NHANES also have significantly higher mean lymphocyte counts vs. U.S.-born participants. Overweight and obese participants have significantly higher mean lymphocyte counts as compared with normal weight participants. Current smokers also have significantly higher mean lymphocyte counts as compared with those who had never smoked.

On average, monocyte counts are significantly lower in non-Hispanic Blacks as compared with those in non-Hispanic Whites. Additionally, females have a lower mean monocyte count vs. that of males. Non-U.S.-born NHANES participants have significantly lower monocyte counts. However, the mean monocyte counts for overweight and obese members of the U.S. adult population are significantly higher than the mean monocyte count for normal weight participants. Current and former smokers both have significantly higher mean monocyte counts vs. those who have never smoked.

On average, non-Hispanic Blacks have significantly lower neutrophil counts vs. non-Hispanic Whites. Females, on average, have significantly higher neutrophil counts vs. males. Participants aged 30–79, on average, have significantly lower neutrophil counts vs. participants aged 20–29. Neutrophil counts are significantly lower for non-U.S.-born participants. Neutrophil counts are significantly higher for overweight and obese NHANES participants. Former and current smokers both have significantly higher mean neutrophil counts vs. those who had never smoked.

Eosinophil counts are significantly lower in non-Hispanic Blacks vs. non-Hispanic Whites. On average, females also have significantly lower eosinophil counts as compared with males and those born outside of the USA have significantly higher eosinophil counts as compared with participants declaring themselves as U.S. born. Additionally, overweight and obese NHANES participants have significantly higher mean eosinophil counts vs. normal weight participants. Likewise, former and current smokers have significantly higher mean eosinophil counts vs. never smokers.

Basophil counts, on average, are significantly lower in non-Hispanic Blacks vs. non-Hispanic Whites. Participants aged 20–29 have a significantly lower mean basophil count vs. those of participants in the age groups 30–59, 60–79, and 80 and older. Mean basophils counts are significantly higher in obese NHANES participants as compared to normal-weight NHANES participants. Additionally, current smokers have significantly higher basophil counts vs. those who had never smoked.

On average, platelet counts are significantly higher in Hispanics and others as compared with non-Hispanic Whites. Females also have significantly higher platelet counts than males. Participants aged 20–29 have a significantly higher mean platelet count vs. those of participants in the age groups 30–59, 60–79, and 80 and older. Participants who are overweight and obese have significantly higher mean platelet counts as compared with participants with normal weight. Current smokers also have a significantly higher mean platelet count as compared with those who had never smoked. However, participants born outside of the United States have a significantly lower mean platelet count as compared with U.S.-born participants.

The mean NLR value for non-Hispanic Blacks is significantly lower as compared with non-Hispanic Whites. On average, NLR values are also significantly lower in non-U.S.-born participants as compared with U.S.-born participants. Participants aged 20–29 have a significantly higher mean NLR value vs. those of participants in the age groups 30–59, 60–79, and 80 and older. Additionally, current smokers and former smokers have significantly higher mean NLR values as compared with that of those who had never smoked.

The mean PLR value for non-Hispanic Blacks is significantly lower as compared with non-Hispanic Whites. Participants aged 20–29 have a significantly lower mean PLR value vs. that of participants in the age groups 30–59, 60–79, and 80 and older. Additionally, current smokers have a significantly higher mean PLR value as compared with those who had never smoked. Participants in the obese category also had a higher mean PLR value as compared with normal weight participants.

## 4. Discussion

NLR and PLR values have been used as predictors of mortality in patients with various types of cancers, acute coronary syndrome, and other chronic inflammatory states [1,2,3,4,5,6]. These markers have also been reported to predict mortality rates of patients affected by the novel coronavirus [23,24]. While there is a growing body of research on the prognostic power of NLR, PLR, and associated white blood cell counts in regard to disease progression and outcomes, not much is known about contributors of blood cellular variations in the general, healthy population. There are some studies that have tried to define a range of normal values but were only investigated with a small study cohort and, moreover, were not looked at across various demographic and behavioral factors. The present study analyzed a large U.S. dataset of over 16,000 participants and reported the mean values of lymphocyte count, monocyte count, segmented neutrophil count, eosinophil count, basophil count, platelet count, NLR, and PLR for the general, healthy population and stratified values by various demographic and behavioral factors. NLR and PLR values were found to vary significantly with race, age, and smoking status. The mean NLR value in the non-Hispanic White population is 2.27, while the non-Hispanic Black population has a mean NLR value of 1.78. The national weighted average PLR value in the non-Hispanic White population is 123.12, while the non-Hispanic Black population has a national weighted average PLR value of 119.77. Participants born outside of the USA were also found to have significantly lower NLR values than U.S.-born participants. These findings have been replicated in other epidemiological studies that have examined inflammatory markers among Latinos, and have been partially explained by differences in behavioral risk factors and acculturative stress [25,26]. Participants aged 20–29 had a mean NLR value of 2.06, while the age groups 30–59, 60–79, and over 80 had mean NLR values of 2.13, 2.35, and 2.81, respectively. Participants aged 20–29 had a mean PLR value of 114.92, while the age groups 30–59, 60–79, and over 80 had mean PLR values of 121.67, 128.62, and 133.78, respectively. Smoking status endorsed a significant difference in mean NLR values in which non-smokers had a mean NLR value of 2.1, current smokers had a mean NLR value of 2.22, and former smokers had a mean NLR value of 2.28. Smoking status also endorsed a significant difference in mean PLR values in which non-smokers had a mean PLR value of 123.51, current smokers had a mean PLR value of 111.02, and former smokers had a mean PLR value of 124.77.

Generally, a higher NLR value and a higher PLR value have been correlated with high mortality and poor prognosis in non-healthy populations [27,28,29,30]. However, in the general, healthy population there are not yet standardized values for a normal range of NLR and PLR values that consider various modifiable and nonmodifiable factors. In this study, it was found that the mean NLR and PLR values differed by factors such as race, age, and smoking status. The results showed that non-Hispanic Blacks had the lowestmean NLR value of all racial groups, which was consistent with previous studies [19]. A higher prevalence of benign ethnic neutropenia in populations of African descent may explain the lower mean NLR value in non-Hispanic Blacks but further investigation is needed [31,32].

Participants aged 20–29, on average, had lower NLR and PLR values than participants older than age 30. Higher NLR and PLR values in older populations may be attributed to multiple causes. One such cause is the complex process of immunosenescence, i.e., the age-related decline of the immune system. Immunosenescence can cause decreased production of white blood cells such as lymphocytes, neutrophils, monocytes, and regulatory B and T cells as reflected by age-related response to inflammation, including increased susceptibility to infections, varied responses to vaccines and immunomodulators, and increased prevalence of chronic inflammatory states or conditions [33]. Altered levels of white blood cell production would directly affect NLR and PLR values, thus, explaining the higher range of NLR and PLR values for older age groups.

Smoking status was found to be significantly associated with NLR values, in which non-smokers were found to have the lowest NLR levels as compared with current and former smokers. Smoking status was also found to be significantly associated with PLR values, in which current smokers were found to have the lowest PLR levels as compared with non-smokers and former smokers. These findings are consistent with previous studies, that establish NLR increases with increasing pack-years [34] and PLR decreases with increasing pack-years [21]. Increased NLR values in current and former smokers can possibly be explained by the changes in white blood cell counts that are caused by gaseous and particulate cigarette smoke. Decreased PLR values in current smokers are also due mostly to cigarette smoke causing changes in white blood cell counts, since current smoking is known to be correlated with thrombogenic effects, likely related to increased platelet counts and enhanced platelet function [35]. Direct activation of epithelial and immune cells in the oral and conducting airways induces the secretion of proinflammatory factors such as IL-8 and TNF-alpha and recruitment of white blood cells such as neutrophils that can potentially modulate NLR and PLR levels and cause chronic inflammation [36].

In general, inflammation has been implicated as a causative factor or major contributor to morbidity in an increasing number of chronic conditions. Aging and smoking, as discussed above, are two common causes of increased inflammation. COVID-19 also causes an acute hyperinflammatory state, which, if prolonged, can cause widespread damage to multiple organ systems [24], contributing to the increasingly recognized long COVID syndrome. However, one of the most recognized causes of inflammation is malignancy. Tumors can release systemic cytokines that predispose the body to developing many inflammatory sequelae, including atherosclerosis, thrombosis, and paraneoplastic manifestations [23]. These inflammatory phenomena are associated with worse prognosis in cancer patients and can also lead to eventual increased risk for cardiovascular and cerebrovascular disease [29]. Indeed, the inflammatory marker high sensitivity C-reactive protein is already used to screen for coronary disease predisposed by chronic low-level inflammation. The NLR and PLR, as other markers of inflammation, can serve as adjunctive markers to guide clinicians and their patients about the treatment, prognosis, and counseling in the course of cancer.

The current study has several strengths and limitations. One major strength is the use of a large, nationally representative non-institutionalized sample of US residents. This comprehensive dataset allows examination of natural contributors of blood cellular variations across various factors to be able to derive statistically significant differences in NLR and PLR values in different groups of people. Some limitations include the exclusion of patients with any chronic inflammatory conditions and as a result may have caused an underestimation of inflammatory marker levels in the general population. Another limitation, and possible area of further research, is the correlation of NLR and PLR values with other well-known markers of inflammation, such as C-Reactive protein, which may give further insight into how overall inflammation is modulated by demographic and behavioral factors.

## 5. Conclusions

In conclusion, the present analysis of a large U.S. dataset of over 16,000 subjects reports the mean values of neutrophil-to-lymphocyte and platelet-to-lymphocyte ratios in a healthy, general population, using various demographic and behavioral factors. The NLR and PLR values significantly varied with race, age, nativity, and smoking status. It was found that non-Hispanic Blacks, older people, people born in the USA, and people who have a current or past smoking history had higher NLR values. The differences in inflammatory markers by nativity highlight the need to better uncover the biobehavioral mechanisms and pathways linking acculturation with health outcomes. These findings have important clinical implications because they indicate the need to set different cutoff points by race, age, and sex for predictive markers using in risk assessment of various illnesses.

## Figures and Tables

**Table 1:** Sociodemographic and clinical characteristics of the NHANES sample: 2009–2016.

Characteristics of Interest ¹		Unweighted Sample	Weighted Population
Race	Non-Hispanic White	6177 (36.7)	62.46 (58.54–66.22)
	Non-Hispanic Black	3493 (20.7)	11.65 (9.949–13.6)
	Hispanic	4615 (27.4)	16.7 (13.99–19.8)
	Other	2564 (15.2)	9.2 (8.01–10.53)
Sex	Male	8550 (50.7)	50.47 (49.86–51.26)
	Female	8299 (49.3)	49.53 (48.74–50.32)
Age (years)	20–29	3822 (22.7)	24.63 (23.1–26.23)
	30–59	9366 (55.6)	58.86 (57.41–60.29)
	60–79	3025 (18.0)	14.02 (13.15–14.94)
	80+	636 (3.8)	2.49 (2.2–2.8)
Education	<High School	3902 (23.2)	15.62 (14.1–17.27)
	High School/General Educational Development	3636 (21.6)	20.81 (19.62–22.06)
	Some College, or Associate of Arts	4997 (29.7)	31.72 (30.29–33.18)
	>College Graduate	4291 (25.5)	31.85 (29.52–34.28)
Nativity	United States born	11,042 (65.5)	78.83 (76.49–80.99)
	Non-United States born	5797 (34.4)	21.17 (19.01–23.51)
Body mass index (Kg/m^2^)	Underweight (<18.5)	291 (1.7)	1.791(1.527–2.098)
	Normal weight (18.5–24.9)	4999 (29.7)	31.43 (29.89–33.01)
	Overweight (25.0–29.9)	5369 (31.9)	33.75 (32.68–34.83)
	Obese (>30)	5404 (32.1)	33.03 (31.7–34.39)
Smoking status	Non-smoker	10,107 (60.0)	59.63 (58.06–61.18)
	Current smoker	3454 (20.5)	19.74 (18.73–20.78)
	Former smoker	3272 (19.4)	20.63 (19.36–21.96)
Alcohol usage	Non-drinker	2133 (12.7)	11.61 (10.29–13.07)
	Moderate drinker	4807 (28.5)	32.83 (31.5–34.19)
	Heavy drinker	7406 (44.0)	55.56 (54.14–56.97)
Lymphocytes (1000 cells/µL)		2.17 (2.15–2.19)	2.14 (2.11–2.16)
Monocytes (1000 cells/µL)		0.55 (0.54–0.55)	0.56 (0.55–0.56)
Segmented neutrophils (1000 cells/µL)		4.23 (4.2–4.27)	4.28 (4.23–4.34)
Eosinophils (1000 cells/µL)		0.2 (0.2–0.2)	0.20 (0.19–0.20)
Basophils number (1000 cells/µL)		0.05 (0.04–0.05)	0.05 (0.04–0.05)
Platelet count (1000 cells/µL)		239.46 (238.45–240.47)	238.89 (237.26–240.53)
Neutrophil/lymphocyte ratio (NLR)		2.12 (2.10–2.14)	2.16 (2.13–2.19)
Platelet/lymphocyte ratio (PLR)		120.19 (119.48–120.91)	121.31 (120.01–122.61)

^1^ We summarized the data using unweighted mean (95% CI), unweighted count (percent), weighted mean (95% CI), and weighted proportion (95% CI).

**Table 2:** Univariate associations of sociodemographic characteristics with clinical inflammatory markers: NHANES 2009–2016.

Sociodemographic Characteristics ^2^		Lymphocytes (1000 Cells/µL)	Monocytes (1000 Cells/µL)	Segmented Neutrophils (1000 Cells/µL)
Race	Non-Hispanic White	2.09 (2.05–2.12)	0.57 (0.56–0.58)	4.37 (4.30–4.44)
	Non-Hispanic Black	2.24 (2.20–2.27)	0.52 (0.51–0.53)	3.66 (3.58–3.74)
	Hispanic	2.26 (2.20–2.29)	0.55 (0.54–0.56)	4.44 (4.36–4.53)
	Other	2.15 (2.12–2.19)	0.52 (0.51–0.53)	4.13 (4.03–4.23)
Sex	Male	2.09 (2.06–2.13)	0.58 (0.57–0.59)	3.95 (3.92–3.97)
	Female	2.18 (2.16–2.21)	0.54 (0.54–0.55)	4.11 (4.08–4.14)
Age (years)	20–29	2.27 (2.23–2.30)	0.56 (0.55–0.57)	4.38 (4.28–4.47)
	30–59	2.14 (2.11–2.17)	0.55 (0.54–0.55)	4.27 (4.22–4.33)
	60–79	1.94 (1.90–1.99)	0.57 (0.55–0.59)	4.13 (4.04–4.21)
	80+	1.94 (1.69–2.19)	0.62 (0.60–0.64)	4.36 (4.17–4.55)
Education	<High School	2.23 (2.20–2.27)	0.56 (0.55–0.58)	4.46 (4.36–4.56)
	High School/General Educational Development	2.20 (2.16–2.25)	0.57 (0.56–0.59)	4.45 (4.37–4.52)
	Some College, or Associate of Arts	2.17 (2.14–2.20)	0.56 (0.55–0.58)	4.35 (4.28–4.43)
	>College Graduate	2.02 (1.20–2.05)	0.53 (0.52–0.54)	4.01 (3.94–4.09)
Nativity	United States born	2.12 (2.10–2.15)	0.56 (0.55–0.57)	4.34 (4.28–4.40)
	Non-United States born	2.19 (2.17–2.21)	0.53 (0.52–0.54)	4.08 (4.01–4.14)
Body mass index (Kg/m^2^)	Underweight (<18.5)	2.09 (1.99–2.19)	0.52 (0.49–0.54)	4.06 (3.77–4.33)
	Normal weight (18.5–24.9)	2.01 (1.99–2.02)	0.53 (0.52–0.53)	3.40 (3.92–3.07)
	Overweight (25.0–29.9)	2.12 (2.08–2.15)	0.56 (0.55–0.56)	4.18 (4.10–4.25)
	Obese (>30)	2.28 (2.25–2.31)	0.58 (0.57–0.59)	4.66 (4.59–4.72)
Smoking status	Non-smoker	2.09 (2.07–2.11)	0.54 (0.53–0.54)	4.09 (4.04–4.15)
	Current smoker	2.36 (2.32–2.42)	0.60 (0.59–0.60)	4.87 (4.77–4.97)
	Former smoker	2.04 (2.01–2.07)	0.57 (0.56–0.57)	4.26 (4.17–4.36)
Alcohol usage	Non-drinker	2.16 (2.10–2.22)	0.54 (0.53–0.56)	4.17 (4.07–4.27)
	Moderate drinker	2.08 (2.04–2.12)	0.54 (0.53–0.55)	4.19 (4.12–4.27)
	Heavy drinker	2.16 (2.13–2.18)	0.57 (0.56–0.58)	4.34 (4.28–4.41)
**Sociodemographic Characteristics**		**Eosinophils (1000 Cells/µL)**	**Basophils (1000 Cells/µL)**	**Platelet/Lymphocyte Ratio**
Race	Non-Hispanic White	0.20 (0.19–0.20)	0.05 (0.45–0.05)	123.12 (121.13–125.11)
	Non-Hispanic Black	0.18 (0.17–0.19)	0.04 (0.04–0.05)	119.77 (117.49–122.06)
	Hispanic	0.20 (0.20–0.21)	0.04 (0.04–0.05)	116.33 (114.69–117.97)
	Other	0.21 (0.20–0.22)	0.04 (0.04–0.05)	119.84 (116.88–122.81)
Sex	Male	0.24 (0.24–0.24)	0.04 (0.04–0.05)	117.75 (116.25–119.26)
	Female	0.20 (0.20–0.20)	0.04 (0.04–0.05)	124.94 (123.28–126.61)
Age (years)	20–29	0.19 (0.19–0.20)	0.04 (0.04–0.05)	114.92 (113.02–116.83)
	30–59	0.19 (0.19–0.20)	0.05 (0.04–0.05)	121.67 (120.17–123.18)
	60–79	0.21 (0.20–0.22)	0.05 (0.05–0.05)	128.62 (125.83–131.42)
	80+	0.22 (0.20–0.23)	0.05 (0.04–0.05)	133.78 (127.83–139.73)
Education	<High School	0.21 (0.20–0.22)	0.05 (0.04–0.05)	115.05 (112.75–117.35)
	High School/General Educational Development	0.20 (0.19–0.21)	0.05 (0.05–0.05)	118.93 (116.61–121.24)
	Some College, or Associate of Arts	0.19 (0.19–0.20)	0.05 (0.05–0.05)	121.08 (119.24–122.91)
	>College Graduate	0.18 (0.18–0.19)	0.04 (0.04–0.04)	126.11 (124.03–128.19)
Nativity	United States born	0.19 (0.19–0.20)	0.05 (0.05–0.05)	122.78 (121.22–124.33)
	Non-United States born	0.20 (0.20–0.21)	0.04 (0.04–0.04)	115.85 (114.17–117.52)
Body mass index (Kg/m^2^)	Underweight (<18.5)	0.17 (0.15–0.19)	0.05 (0.04–0.06)	124.61 (118.82–130.40)
	Normal weight (18.5–24.9)	0.18 (0.17–0.18)	0.04 (0.04–0.04)	125.01 (122.93–127.09)
	Overweight (25.0–29.9)	0.20 (0.19–0.20)	0.04 (0.04–0.05)	120.21 (118.34–122.08)
	Obese (>30)	0.21 (0.21–0.22)	0.05 (0.05–0.05)	118.56 (116.79–120.32)
Smoking status	Non-smoker	0.18 (0.18–0.19)	0.04 (0.04–0.04)	123.51 (122.08–124.94)
	Current smoker	0.22 (0.22–0.23)	0.06 (0.05–0.06)	111.02 (108.89–113.16)
	Former smoker	0.20 (0.20–0.21)	0.05 (0.04–0.05)	124.77 (122.55–127.00
Alcohol usage	Non-drinker	0.19 (0.18–0.20)	0.04 (0.38–0.05)	122.41 (119.53–125.29)
	Moderate drinker	0.19 (0.18–0.20)	0.04 (0.04–0.05)	124.21 (122.17–126.25)
	Heavy drinker	0.20 (0.20–0.21)	0.05 (0.05–0.05)	119.87 (118.14–121.60)
**Sociodemographic Characteristics**		**Platelet Count (1000 Cells/µL)**	**Neutrophil/Lymphocyte Ratio**	
Race	Non-Hispanic White	235.64 (233.72–237.56)	2.27 (2.22–2.31)	
	Non-Hispanic Black	243.92 (240.75–247.09)	1.78 (1.74–1.83)	
	Hispanic	245.18 (242.85–247.50)	2.10 (2.04–2.16)	
	Other	240.52 (236.65–244.39)	2.03 (1.98–2.08)	
Sex	Male	243.37 (242.34–244.40)	2.19 (2.15–2.23)	
	Female	263.52 (262.50–264.54)	2.14 (2.10–2.17)	
Age (years)	20–29	242.92 (240.37–245.46)	2.06 (2.01–2.11)	
	30–59	240.68 (238.76–242.60)	2.13 (2.1–2.17)	
	60–79	227.22 (223.46–230.98)	2.35 (2.29–2.41)	
	80+	211.45 (206.22–216.68)	2.81 (2.64–2.97)	
Education	<High School	238.89 (235.65–242.13)	2.16 (2.09–2.22)	
	High School/General Educational Development	239.56 (236.47–242.65)	2.19 (2.14–2.24)	
	Some College, or Associate of Arts	241.24 (239.15–243.33)	2.17 (2.12–2.21)	
	>College Graduate	235.18 (232.56–237.81)	2.15 (2.09–2.20)	
Nativity	United States born	239.20 (237.44–240.94)	2.21 (2.17–2.25)	
	Non-United States born	236.40 (233.73–239.07)	1.98 (1.95–2.02)	
Body mass index (Kg/m^2^)	Underweight (<18.5)	237.38 (227.95–246.81)	2.13 (1.95–2.31)	
	Normal weight (18.5–24.9)	232.19 (230.01–234.37)	2.14 (2.09–2.20)	
	Overweight (25.0–29.9)	233.71 (231.61–235.80)	2.14 (2.10–2.18)	
	Obese (>30)	249.83 (247.43–252.24)	2.19 (2.15–2.23)	
Smoking status	Non-smoker	239.27 (237.45–241.08)	2.1 (2.07–2.13)	
	Current smoker	241.36 (238.94–243.78)	2.22 (2.17–2.28)	
	Former smoker	234.08 (231.28–236.87)	2.28 (2.23–2.34)	
Alcohol usage	Non-drinker	242.17 (238.08–246.26)	2.1 (2.03–2.15)	
	Moderate drinker	237.01 (234.20–239.81)	2.2 (2.15–2.25)	
	Heavy drinker	238.37 (236.25–240.5)	2.17 (2.13–2.20)	

^2^ We present group means (95% CI) of inflammatory markers for each level of the predictors of interest.

**Table 3:** Multivariable analyses of inflammatory markers of the U.S. population: NHANES 2009–2016.

Sociodemographic Characteristics		Lymphocytes (1000 Cells/µL)	Monocytes (1000 Cells/µL)	Segmented Neutrophils (1000 Cells/µL)
		Estimate (95% CI)	Estimate (95% CI)	Estimate (95% CI)
Race	Non-Hispanic White	REF	REF	REF
	Non-Hispanic Black	0.058 (0.014–0.102) *	−0.055 (−0.066–−0.044) ***	−0.852 (−0.960–−0.745) ***
	Hispanic	0.0683 (0.017–0.120) *	−0.012 (−0.027–−0.002)	0.080 (−0.051–0.213)
	Other	0.060 (−0.005–0.125)	−0.023 (−0.039–−0.007) **	0.053 (−0.084–0.190)
Sex	Male	REF	REF	REF
	Female	0.116 (0.080–0.153) ***	−0.047 (−0.056–−0.037) ***	0.053 (0.165–0.318) ***
Age (years)	20–29	REF	REF	REF
	30–59	−0.145 (−0.188–−0.103) *	−0.016 (−0.028–−0.005) **	−0.123 (−0.211–−0.344) **
	60–79	−0.312 (−0.370–−0.253) *	0.002 (−0.018–0.023)	−0.252 (−0.353–−0.150) ***
	80+	−0.228 (−0.514–0.058)	0.062 (0.370–0.087) ***	0.062 (−0.157–0.281)
Education	<High School	REF	REF	REF
	High School/General Educational Development	0.002 (−0.046–0.050)	0.001 (−0.141–0.016)	−0.816 (−0.203–0.040)
	Some College, or Associate of Arts	−0.242 (−0.783–0.030)	0.001 (−0.012–0.140)	−0.159 (−0.289–−0.029) *
	>College Graduate	−0.095 (−0.147–−0.043) ***	−0.027 (−0.038–−0.007) **	−0.331 (−0.454–−0.209) ***
Nativity	United States born	REF	REF	REF
	Non-United States born	0.063 (0.023–0.104) **	−0.016 (−0.029–−0.003) **	−0.234 (−0.341–−0.127) ***
Body mass index (Kg/m^2^)	Underweight (<18.5)	−0.012 (−0.117–0.093)	−0.004 (−0.029–0.021)	−0.034 (−0.334–0.267)
	Normal weight (18.5–24.9)	REF	REF	REF
	Overweight (25.0–29.9)	0.139 (0.096–0181) ***	0.025 (0.015–0.034) ***	0.237 (0.145–0.329) ***
	Obese (>30)	0.286 (0.241–0.330) ***	0.055 (0.044–0.066) ***	0.720 (0.632–0.809) ***
Smoking status	Non-smoker	REF	REF	REF
	Current smoker	0.282 (0.231–0.333) ***	0.054 (0.043–0.065) ***	0.756 (0.662–0.850) ***
	Former smoker	0.005 (−0.038–0.049)	0.016 (0.003–0.029) **	0.100 (0.008–0.191) *
Alcohol usage	Non-drinker	REF	REF	REF
	Moderate drinker	−0.011 (−0.093–0.070)	−0.015 (−0.031–0.001)	0.025 (−0.075–0.013)
	Heavy drinker	−0.200 (−0.091–0.051)	−0.003 (−0.17–0.012)	0.015 (−0.072–0.103)
**Sociodemographic Characteristics**		**Eosinophils (1000 Cells/µL)**	**Basophils (1000 Cells/µL)**	
		**Estimate (95% CI)**	**Estimate (95% CI)**	
Race	Non-Hispanic White	REF	REF	
	Non-Hispanic Black	−0.018 (−0.027–−0.010) ***	−0.005 (−0.009–−0.002) **	
	Hispanic	−0.003 (−0.0130–0.007)	−0.002 (−0.007–0.002)	
	Other	0.016 (0.002–0.030) *	−0.001 (−0.005–0.004)	
Sex	Male	REF	REF	
	Female	−0.020 (−0.028–−0.012) ***	0.002 (−0.001–0.005)	
Age (years)	20–29	REF	REF	
	30–59	−0.001 (−0.010–0.008)	0.004 (0.001–0.006) *	
	60–79	0.011 (−0.002–0.024)	0.007 (0.002–0.011) **	
	80+	0.032 (0.012–0.053) **	0.010 (0.003–0.017) **	
Education	<High School	REF	REF	
	High School/General Educational Development	−0.007 (−0.021–0.007)	−0.001 (−0.004–0.003)	
	Some College, or Associate of Arts	−0.009 (−0.020–0.003)	−0.001 (−0.005–0.003)	
	>College Graduate	−0.011 (−0.023–0.001)	−0.002 (−0.005–0.001) *	
Nativity	United States born	REF	REF	
	Non-United States born	0.010 (0.001–0.020) **	−0.002 (−0.005–0.001)	
Body mass index (Kg/m^2^)	Underweight (<18.5)	−0.003 (−0.027–0.020)	0.003 (−0.006–0.127)	
	Normal weight (18.5–24.9)	REF	REF	
	Overweight (25.0–29.9)	0.018 (0.009–0.027) ***	0.003 (−0.001–0.006)	
	Obese (>30)	0.036 (0.027–0.046) ***	0.011 (0.008–0.015) ***	
Smoking status	Non-smoker	REF	REF	
	Current smoker	0.039 (0.029–0.049) ***	0.034 (0.010–0.018) ***	
	Former smoker	0.012 (0.001–0.022) *	0.002 (−0.002–0.005)	
Alcohol usage	Non-drinker	REF	REF	
	Moderate drinker	−0.002 (−0.013–0.009)	0.002 (−0.003–0.006)	
	Heavy drinker	0.030 (−0.008–0.014)	0.004 (−0.001–0.009)	
**Sociodemographic Characteristics**		**Platelet Count**	**Neutrophil/Lymphocyte Ratio**	
		**Estimate (95% CI)**	**Estimate (95% CI)**	
Race	Non-Hispanic White	REF	REF	
	Non-Hispanic Black	3.404 (−0.383–7.190)	−0.445 (−0.517–−0.372) ***	
	Hispanic	10.682 (7.000–14.370) ***	−0.030 (−0.0136–0.076)	
	Other	11.102 (5.512–16.690) ***	−0.0653 (−0.0156–0.025)	
Sex	Male	REF	REF	
	Female	27.174 (25.060–29.289) ***	−0.029 (−0.074–0.0153)	
Age (years)	20–29	REF	REF	
	30–59	−2.709 (−3.215–4.968)	0.090 (0.031–0.150) **	
	60–79	−15.183 (−19.935–−10.429) ***	0.278 (0.204–0.351) ***	
	80+	−25.420 (−31.748–−19.094) ***	0.707 (−0.162–0.038) ***	
Education	<High School	REF	REF	
	High School/General Educational Development	0.876 (−3.215–4.968)	−0.034 (−0.0121–0.052)	
	Some College, or Associate of Arts	−0.157 (−4.287–3.972)	−0.0470 (−0.0133–0.049)	
	>College Graduate	−2.177 (−6.894–2.540)	−0.062 (−0.016–0.038)	
Nativity	United States born	REF	REF	
	Non-United States born	−7.830 (−11.931–−3.729) ***	−0.186 (−0.258–−0.113) ***	
Body mass index (Kg/m^2^)	Underweight (<18.5)	−2.310 (−12.510–7.888)	−0.0262 (−0.0232–0.179)	
	Normal weight (18.5–24.9)	REF	REF	
	Overweight (25.0–29.9)	5.643 (2.840–8.446) ***	−0.110 (−0.171–−0.049) ***	
	Obese (>30)	18.059 (14.911–21.207) ***	−0.034 (−0.099–0.031)	
Smoking status	Non-smoker	REF	REF	
	Current smoker	3.908 (0.739–7.077) *	0.11 (0.05–0.171) ***	
	Former smoker	0.600 (−2.494–3.634)	0.076 (0.018–134) *	
Alcohol usage	Non-drinker	REF	REF	
	Moderate drinker	−1.433 (−6.490–3.624)	2.27 (2.038–2.508) ***	
	Heavy drinker	0.847 (−3.783–5.477) ***		

* *p* < 0.05, ** *p* < 0.01, *** *p* < 0.001; REF refers to referent group.

## Data Availability

Data supporting these results are from the publicly available National Health and Nutrition Examination Survey. Datasets, questionnaires, and related documentation can be found at https://wwwn.cdc.gov/nchs/nhanes (accessed on 1 August 2022).
